# Finite element analysis of the tibial bone graft in cementless total knee arthroplasty

**DOI:** 10.1186/s13018-018-0830-1

**Published:** 2018-05-16

**Authors:** Koji Totoribe, Etsuo Chosa, Go Yamako, Hiroaki Hamada, Koki Ouchi, Shutaro Yamashita, Gang Deng

**Affiliations:** 1 Department of Orthopaedic Surgery, Faculty of Medicine, University of Miyazaki, 5200 Kihara, Kiyotake, Miyazaki, 889-1692 Japan; 2 Department of Mechanical Design Systems, Faculty of Engineering, University of Miyazaki, 1-1 Gakuen Kibana-dai-nishi, Miyazaki, 889-2192 Japan

**Keywords:** Total knee arthroplasty, Bone graft, Finite element analysis

## Abstract

**Background:**

Achieving stability of the tibial implant is essential following cementless total knee arthroplasty with bone grafting. We investigated the effects of bone grafting on the relative micromotion of the tibial implant and stress between the tibial implant and adjacent bone in the immediate postoperative period.

**Methods:**

Tibial implant models were developed using a nonlinear, three-dimensional, finite element method. On the basis of a preprepared template, several bone graft models of varying sizes and material properties were prepared.

**Results:**

Micromotion was larger in the bone graft models than in the intact model. Maximum micromotion and excessive stress in the area adjacent to the bone graft were observed for the soft and large graft models. With hard bone grafting, increased load transfer and decreased micromotion were observed.

**Conclusions:**

Avoidance of large soft bone grafts and use of hard bone grafting effectively reduced micromotion and undue stress in the adjacent area.

## Background

Tibial bone defects in total knee arthroplasty (TKA) often are managed with various types of bone grafts, such as solid bone, morselized bone, and combined grafts [1–4]. For optimal results, patients with bone grafts require long-term postoperative therapy. In particular, during weight-bearing gait training, the mechanical condition of the grafted bone and micromotion-preventing fixation of the tibial component influence the degree of micromotion [3]. Whether the patient is able to walk safely immediately postoperatively is an important concern after cementless TKA. The finite element method is being used increasingly for biomechanical analysis of tibial component micromotion. Several studies have analyzed the relative micromotion of implanted prostheses using this method [5–8]. We have previously reported that tibial bone graft combined with cortical bone can be expected to reduce micromotion and stress [8]. However, the simulation was not enough to include the characteristics of implant and bone graft materials in the models or to examine the detailed status of contact and stress distribution while considering micromotion. To our knowledge, no analysis has been conducted considering the actual bone graft size and material properties. We used the finite element method to develop a new precise model of the proximal tibia and tibial implant and meticulously analyzed the biomechanical effects of various types of bone grafts on relative micromotion and stress distribution at the tibial tray–bone interface in the immediate postoperative period.

## Methods

### Intact (I) model

**Fig. 1:**
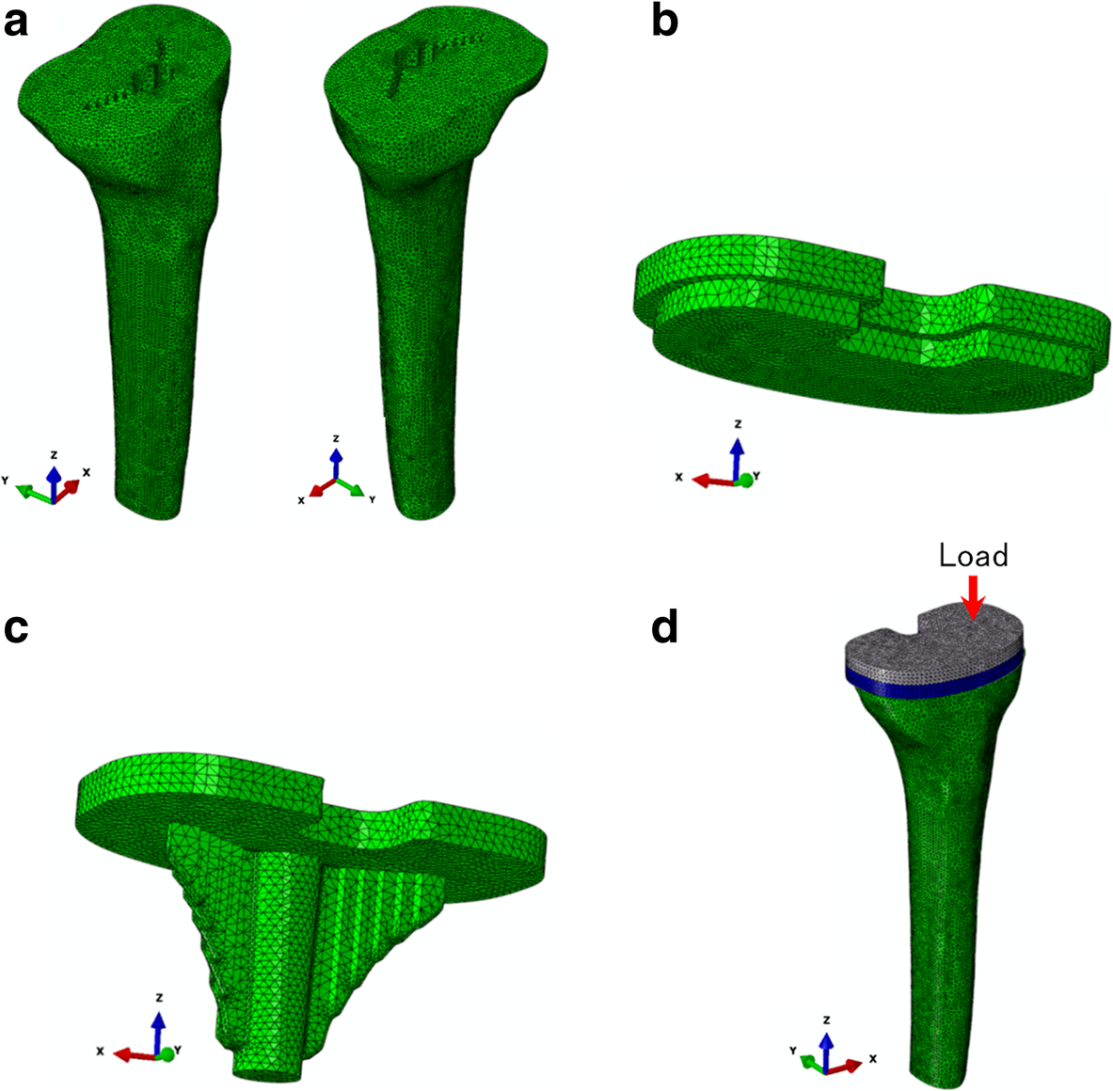
Detailed finite element model of the proximal tibia and tibial component. The directions of the axes of the global coordinate system are shown. **a** Intact tibia showing the contact surface with the tibial component. **b** UHMWPE insert. **c** Tibial baseplate with keel. **d** Load application to the model

To prepare a three-dimensional finite element model, geometric data for the proximal tibia were obtained from 0.6-mm-wide computed tomography (CT) scans of the right tibia of a 40-year-old woman. Before CT scanning, informed consent was obtained from the subject, and the study was approved by our institutional research ethics committee. In the global coordinate system, the long axis of the tibia was the line connecting the center of the ankle joint and the midpoint of the lateral and medial tibial condyles, which was defined as the *z* axis [9]. The transverse plane (*xy*-plane) was perpendicular to the long axis. To simplify the preparation of the models, the fibula was ignored (Fig. 1a). The three-dimensional four-noded tetrahedral element was used for modeling cortical and cancellous bone and tibial baseplate. A tibial model comprising a total of 450,697 solid elements was developed (Fig. 1). For development of the models and their subsequent analysis, the multipurpose finite element analysis software ABAQUS/Standard (Simulia, Tokyo, Japan) was used.

### Tibial component

An appropriate modular tibial baseplate made of cobalt–chrome alloy (#3 size, Osteonics 7000 tibial component; Howmedia Osteonics Corp., Mahwah, NJ, USA), applied in accordance with the manufacturer’s guidelines, was considered in this study (Fig. 1c). The geometry of the modular tibial component was obtained by direct measurement. The tibial insert was composed of 8-mm-thick ultra-high molecular weight polyethylene (UHMWPE). The rounded top surface of the UHMWPE was ignored, and a flat surface was defined (Fig. 1b). The inferior surface of the UHMWPE was attached to the tibial baseplate, which was positioned at the geometric center of the tibia, tilted 3° posteriorly (Fig. 1d).

### Bone graft models

**Fig. 2:**
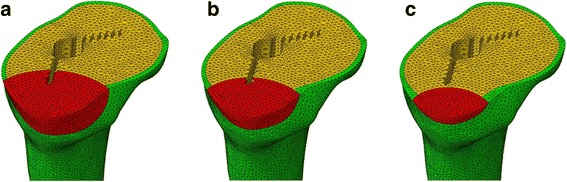
Finite element models of bone grafts. Posterolateral view of the bone graft area. **a** Large, **b** medium, and **c** small bone defects filled with bone graft

Clinically, bone defect locations in the proximal tibia often are distributed in the posteromedial region. Using the standard tibial model, a bone graft model representative of a posteromedially located bone graft was prepared. The models were graded as large, medium, and small in terms of the bone graft depth (9, 6, and 3 mm, respectively; Fig. 2). To account for the various techniques used for bone grafting, two types of bone graft models were prepared in the posteromedial portion, with different material properties. The first was a soft bone graft (Fig. 2a–c) in which the defect was filled with a morselized cancellous bone graft, simulating a mechanical worst-case scenario. The second was a hard bone graft, simulating a stronger bone graft, such as tightly impacted morselized cancellous bone grafts than those achieved with simple morselized cancellous bone. Thus, the bone graft models comprised the following six variations: (1) L–S (large bone graft with soft bone), (2) M–S (medium bone graft with soft bone), (3) S–S (small bone graft with soft bone), (4) L–H (large bone graft with hard bone), (5) M–H (medium bone graft with hard bone), and (6) S–H (small bone graft with hard bone).

### Material properties

For the tibia, Young’s modulus of cortical and cancellous bone was set at 17,000 and 400 MPa, with Poisson’s ratio of 0.30 [10, 11]. Young’s modulus of morselized bone graft for bone graft models has been reported as 42–150 MPa [12–14]. In the present bone graft models, simulating the treatment of bone defects with morselized bone graft, Young’s modulus for the bone graft site was set at 42 or 150 MPa with Poisson’s ratio of 0.2, the respective low- and high-strength bone graft values according to the literature.

The cobalt–chrome alloy used for the tibial baseplate component and the UHMWPE used for the tibial insert were given Young’s modulus (Poisson’s ratio) of 248,000 MPa (0.30) and 667 MPa (0.46), respectively [11]. The interface between the bone and tibial component was treated as a frictional contact problem, with friction set at 0.2 according to the literature [15].

### Boundary and loading conditions

The inferior surface of the distal tibia was fixed in all directions. The maximum loads used in the analyses were the maximum physiological loads used in previously described experiments [16, 17]. A compressive force of approximately three times the body weight, or 1800 N, was used in this study. Regarding the site of loading, the posteromedial portion was used, as in the previously described experiments [5, 18], to simulate a varus alignment. For nonlinear analyses, incremental force control was used in the numerical procedure. Each model was loaded, and the resultant displacement, maximum liftoff, subsidence, and von Mises stress distribution were analyzed and compared.

## Results

For the medial load application shown in Fig. 1d, the stability of the implant and stress distribution are important from the biomechanical viewpoint. Relative micromotion between the tibial tray (Fig. 1c) and tibial bone (Fig. 1a) model at the location of maximum liftoff and at the distal tip of the stem and stress distribution in the tibial bone for the intact and various types of bone graft models shown in Fig. 2 were calculated.

### Displacement and micromotion

#### Displacement

**Fig. 3:**
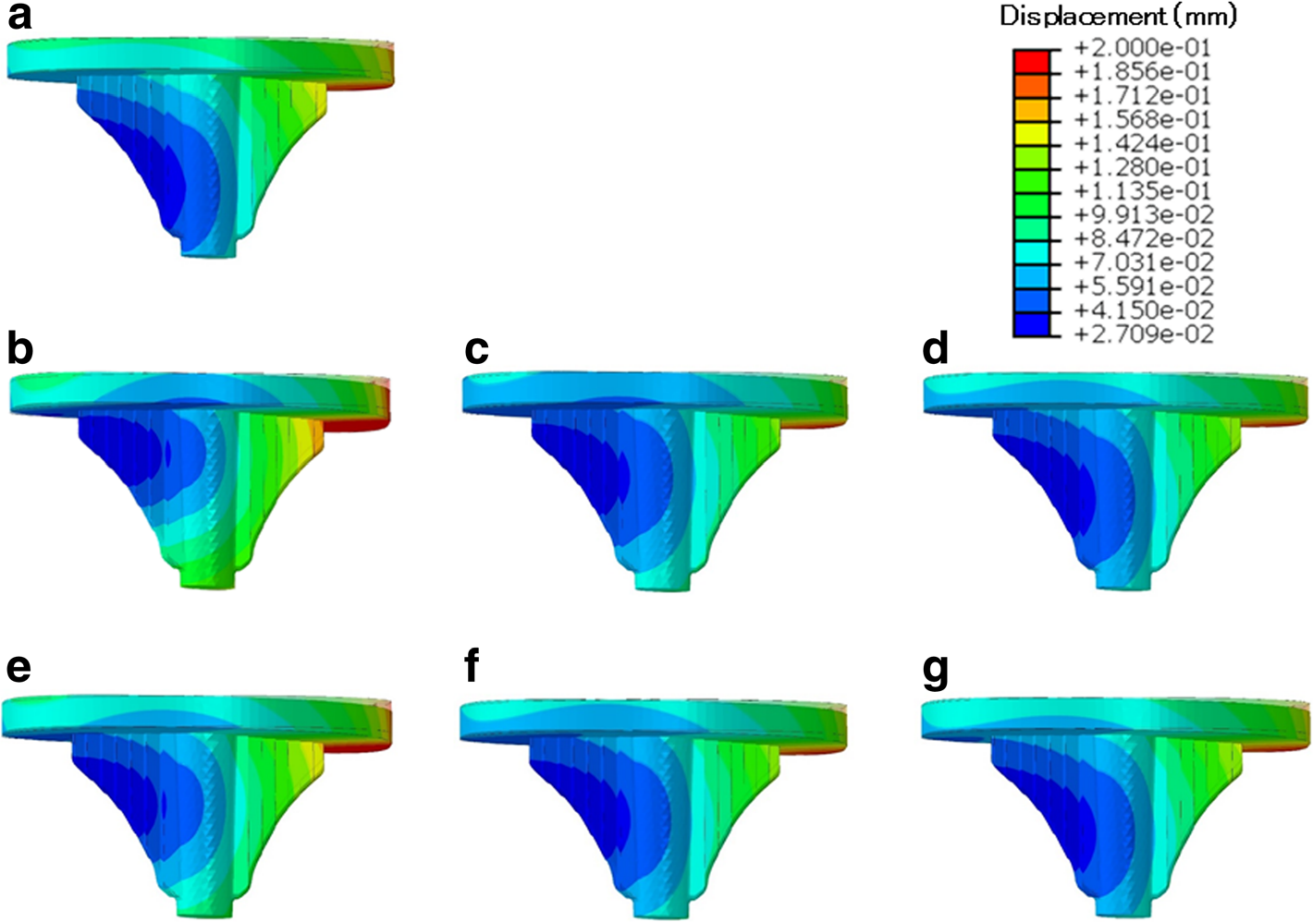
Resultant displacement of the deformed tibial tray under maximum loading in each model. Contour lines of deformation indicate deformation of the tibial tray. Magnification factor of the displacement × 10. **a** I (intact). **b** L–S (large), **c** M–S (medium), and **d** S–S (small) bone grafts with soft bone. **e** L–H (large), **f** M–H (medium), and **g** S–H (small) bone grafts with hard bone

Figure 3 shows the displacement of the tibial tray for all models. Displacement was highest at the periphery of the tibial tray. The medial side of the tibial tray exhibited subsidence at the medial plateau because of the medial load, and the opposite lateral side showed liftoff.

#### Maximum liftoff

**Fig. 4:**
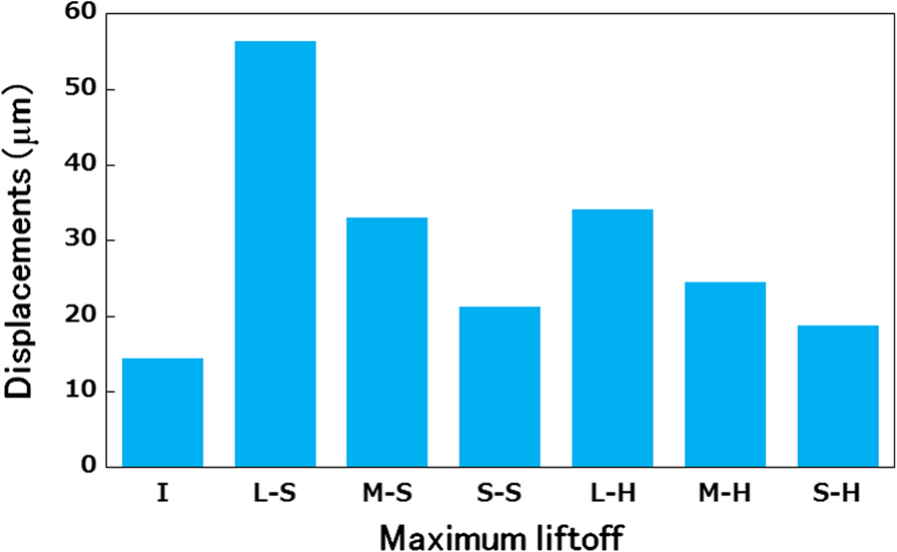
Maximum liftoff of the tibial tray in each model. I (intact), L–S (large), M–S (medium), and S–S (small) bone grafts with soft bone. L–H (large), M–H (medium), and S–H (small) bone grafts with hard bone

Figure 4 shows the relative micromotion (*z*-component) between the tibial tray and tibial bone model at the location of maximum liftoff. The maximum liftoff of the tibial tray in the I model was 14.5 μm. Compared with the I model, all bone graft models showed increased liftoff: 289, 128, and 47% in the L–S (largest motion), M–S, and S–S (least motion) models, respectively.

The maximum liftoff was smaller in the hard than in the soft bone graft models. Comparing the L–S and L–H, M–S and M–H, and S–S and S–H models revealed that the maximum liftoff of the tibial tray was lower in the hard bone graft models by approximately 39, 26, and 12%, respectively, indicating a decrease in the micromotion of hard bone. In the M–H and S–H models, the magnitudes of liftoff were almost identical and smaller than those in the I model.

#### Relative micromotion beneath the stem

**Fig. 5:**
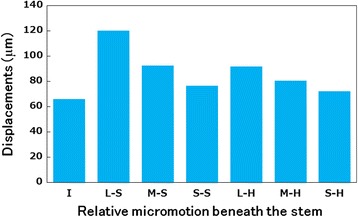
Relative micromotion beneath the stem in each model. I (intact), L–S (large), M–S (medium), and S–S (small) bone grafts with soft bone. L–H (large), M–H (medium), and S–H (small) bone grafts with hard bone

Figure 5 shows the relative micromotion between the tibial tray and tibial bone model at the distal stem tip. The tendency toward micromotion beneath the stem was consistent with that toward liftoff. Micromotion beneath the stem in the I model was 68.8 μm. The increased rate of micromotion beneath the stem was 83, 41, and 16% in L–S (largest motion), M–S, and S–S models (least motion), respectively.

Compared with the soft bone graft models, the magnitudes of micromotion beneath the stem in the hard bone graft models were significantly lower. The L–H and M–H models showed a larger and the S–H model a slightly larger magnitude of micromotion than the I model, demonstrating that hard bone grafts have increased stability.

### Stress distribution

**Fig. 6:**
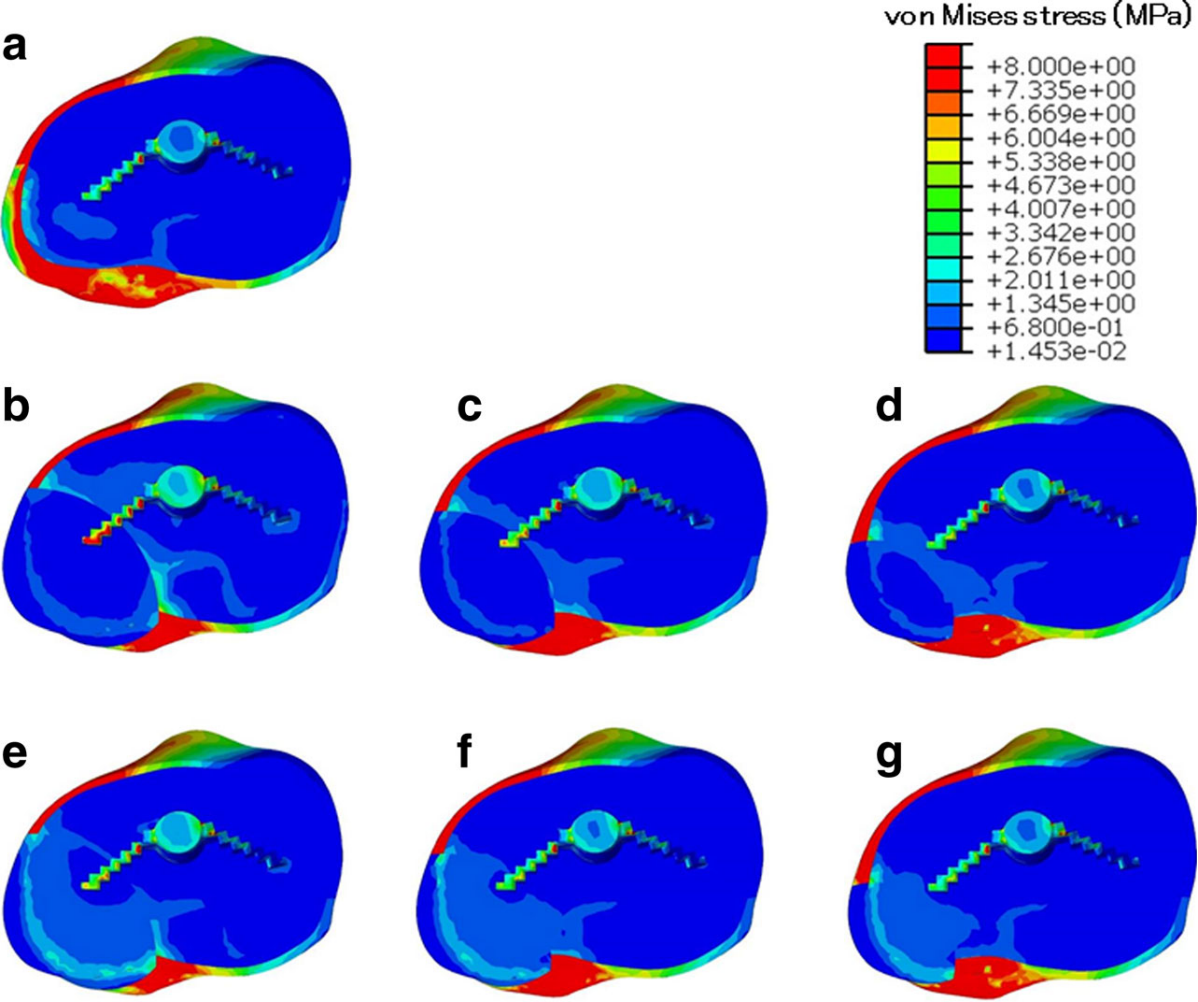
Top view of von Mises stress distribution on the contact surface of the tibia under maximum loading. **a** I (intact). **b** L–S (large bone graft with soft bone). **c** M–S (medium bone graft with soft bone). **d** S–S (small bone graft with soft bone). **e** L–H (large bone graft with hard bone). **f** M–H (medium bone graft with hard bone). **g** S–H (small bone graft with hard bone)

Examination of the overall loading of the I model revealed relatively large von Mises stress values in the posteromedial area of the tibial bone, particularly in the cortical bone at the edge of the point of contact. Lateral aspects of the tibia were less loaded than medial regions, except for the lateral keel of the tibial baseplate (Fig. 6a).

In the soft bone graft models, a stress increase was evident in anteromedial and posteromedial cortical bone and in the medial host cancellous bone region, including in the keel of the tibial baseplate adjacent to the bone graft, relative to the I model (Fig. 6b–d). In particular, the L–S model exhibited high stress in host cancellous bone. In the hard bone graft models, a stress increase was found in the bone graft area, whereas stresses adjacent to the bone graft area were reduced (Fig. 6e–g).

## Discussion

Medial loading resulted in subsidence of the loaded medial tibial plate and liftoff at the periphery on the opposite, unloaded side. The tilting motion of the tibial components observed in this study implies instability of the initial fixation, which could possibly compromise bony ingrowth. The success of cementless implants depends on the ingrowth of bone into the porous coating, which is inhibited by relative micromotion at the implant–bone interface during patient activity [19, 20]. Excess implant micromotion prevents bone formation within porous-surfaced implants. According to Jasty et al. [21], 150 μm of motion results in unstable ingrowth of bone into porous-surfaced implants. Although the 150-μm limit often is quoted in the literature, a range of ± 50 μm was used to assess the sensitivity of the micromotion limit [22]. Our findings showed that soft bone grafting leads to considerable implant micromotion. The relative micromotion observed in the L–S model exceeded 100 μm. This degree of micromotion, if occurring in a clinical situation, may inhibit bony ingrowth and lead to implant loosening. Conversely, use of hard bone decreases micromotion. In all hard bone graft models, the relative micromotion was less than 100 μm. These results suggested that hard bone grafting is necessary in patients requiring a large bone graft.

Loads were transferred via contact between the tibial baseplate and tibial bone. This contact resulted in an increase in the loading of the medial plateau of the tibia. Analysis of the soft bone graft models revealed considerable unloading of the bone graft area concentrated around the bone graft compared with the I model. Tibial host cancellous bone stresses were reduced by hard bone grafting. The L–S model caused high stress in host cancellous bone. High stress levels, particularly acting on weak cancellous bone, have been implicated as the predominant cause of tibial component loosening [23, 24], and the abnormal stress pattern may cause stress shielding in long-term follow-up [25, 26]. The stress pattern produced by load sharing between the implant and bone suggests that large soft bone grafting should be avoided in favor of grafting with hard bone to eliminate excessive stress in host cancellous bone.

In the previous report, the maximum liftoff and relative micromotion were approximately 2–10 times higher than those in this study and suggested that bone grafting causes excessive instability [8]. However, from a modeling viewpoint, the shape of the keel of the tibial baseplate was simple, and the roughness of the keel surface to improve fixation was ignored, treating the interface between the bone and implant as a frictionless contact in the previous report. Furthermore, the graft size and material properties were impractical. However, in this study, the shape of the keel designs was precise, treating the interface between the implant and bone as a frictional contact to provide additional stability, in addition to considering realistic graft size and material properties. We believe that these distinct differences are the main reasons that affected the results.

With regard to the elastic modulus of bone graft materials, several studies have demonstrated that material stiffness and strength vary substantially according to age, diagnosis, and composition of cancellous and corticocancellous bone and that improved fixation can be achieved by impaction during bone grafting [13, 14, 27–29]. In this study, hard bone models showed lower relative micromotion than soft bone grafting models, suggesting that increased stiffness of the bone graft material by impaction led to decreased micromotion and improved the initial fixation of the tibial baseplate. In clinical cases where the quality of host bone varies substantially between patients, the appropriate bone, including impacted bone, should be grafted into bone defects to achieve optimal stability and a good clinical outcome [4, 30, 31].

With respect to eccentric loading, the finite element analysis of bone grafts undertaken in this study represented the worst-case scenario. Our results suggested that grafting defects with hard bone is more likely to yield better fixation especially for larger, posteromedial defects. During surgery, mechanically effective fixation can be achieved by impaction according to the bone graft material used and type of tibial defects. However, this study did not consider changes in mechanical conditions because of bone remodeling, ingrowth, or stress relaxation. Thus, the qualitative trends outlined here offer an approximation of what occurs during the immediate postoperative phase. This study was designed to provide a foundation for a biomechanical rationale that will support selection of treatment. Because the severity of tibial bone defects and range of bone grafts vary in the clinical setting, care should be taken when interpreting these results. Considering these limitations, combining our results with those of previous cadaveric studies may enhance the clinical use of this technique.

## Conclusions

The purpose of this study was to investigate the effects of bone grafting on the relative micromotion of the tibial implant and stress between the tibial implant and adjacent bone. Maximum micromotion and excessive stress in the host bone area adjacent to the bone graft were observed for the soft and large graft models. On the basis of these biomechanical findings, avoidance of large soft bone grafts and use of hard bone grafting will produce better clinical outcomes.

## Abbreviations

CT: Computed tomography
I: Intact
L–H: Large bone graft with hard bone
L–S: Large bone graft with soft bone
M–H: Medium bone graft with hard bone
M–S: Medium bone graft with soft bone
S–H: Small bone graft with hard bone
S–S: Small bone graft with soft bone
TKA: Total knee arthroplasty
UHMWPE: Ultra-high molecular weight polyethylene
